# Targeting casein kinase 2 and ubiquitin-specific protease 7 to modulate RUNX2-mediated osteogenesis in chronic kidney disease

**DOI:** 10.1186/s10020-025-01222-5

**Published:** 2025-05-30

**Authors:** Haifeng Lan, Xiao-Jun Yu, Guangsheng Ling, Yuwei Zeng, Yixi Yang, Meiyang He, Yixiao Yu, Ming Shao

**Affiliations:** 1 Department of Orthopedics, Guangdong Provincial Key Laboratory of Major Obstetric Diseases; Guangdong Provincial Clinical Research Center for Obstetrics and Gynecology; The Third Affiliated Hospital, Guangzhou Medical University, No. 63, Duobao Road, Liwan District, Guangzhou, Guangdong Province 510150 P. R. China; 2 Department of Spine Surgery, Honghui Hospital, Xi’an Jiaotong University, Xi’an, Shaanxi 710054 China; 3 Shaanxi Key Laboratory of Spine Bionic Treatment, Xi’an, Shaanxi 710054 China; 4 The Third School of Clinical Medicine, Guangzhou Medical University, Guangzhou, 511436 P. R. China

**Keywords:** Chronic kidney disease, Mineral bone metabolism disorder, Casein kinase 2, Runt-related transcription factor 2, Ubiquitin-specific protease 7

## Abstract

**Objective:**

Chronic Kidney Disease (CKD) frequently leads to Mineral Bone Disorder (MBD), which significantly affects patient quality of life due to bone fragility and metabolic disturbances. This study investigates the role of Casein Kinase 2 (CK2) and Ubiquitin-Specific Protease 7 (USP7) in modulating Runt-related Transcription Factor 2 (RUNX2)-driven osteogenesis in a CKD-MBD mouse model.

**Methods:**

A CKD-MBD mouse model was established using 5/6 nephrectomy. Bioinformatic analysis of CKD-related datasets identified RUNX2 and USP7 as key genes implicated in bone metabolism. In vivo and in vitro experiments were conducted to assess the effects of CK2-mediated phosphorylation and USP7-induced deubiquitination on RUNX2 stability and function. Histomorphometry, Enzyme-Linked Immunosorbent Assay (ELISA), and micro-CT analyses were performed to evaluate bone density, strength, and metabolic markers.

**Results:**

RUNX2 and USP7 were significantly downregulated in CKD-MBD mice. Silencing RUNX2 impaired osteoblast differentiation, reduced bone density, and increased bone turnover, while CK2 overexpression restored RUNX2 activity by phosphorylation, recruiting USP7 to stabilize RUNX2. Enhanced osteoblast differentiation and improved bone metabolism were observed in CKD-MBD mice upon CK2 activation.

**Conclusion:**

CK2 activation promotes RUNX2 phosphorylation and stabilization by USP7, leading to improved osteogenesis and bone metabolism in CKD-MBD. Targeting the CK2/USP7/RUNX2 axis presents a potential therapeutic strategy for managing CKD-related bone disorders.

**Supplementary Information:**

The online version contains supplementary material available at 10.1186/s10020-025-01222-5.

## Introduction

Chronic kidney disease (CKD) disrupts the balance of mineral metabolism, leading to an imbalance in calcium and phosphorus levels in the body, which in turn results in various mineral bone metabolism disorders (MBDs) such as osteoporosis, osteomalacia, osteopenia, and vascular calcification (Watanabe 2020). These changes in skeletal and mineral metabolism cause skeletal deformities, muscle weakness, fractures, and premature mortality (Bianchi 2020). MBDs are inherent complications of moderate to end-stage CKD (Kalantar-Zadeh et al. 2012). CKD-MBD can be defined as impaired renal function accompanied by imbalances in mineral metabolism and abnormal regulation (Tsuboi et al. 2020). It is a significant factor in the increased incidence of fragility fractures across all age groups (Rysz et al. 2021) and the elevated rates of cardiovascular diseases and mortality related to CKD (Cianciolo et al. 2020). As a syndrome involving biochemistry, skeletal, and cardiovascular pathophysiology (Seifert and Hruska 2016), CKD-MBD is associated with vascular calcification, renal osteodystrophy, and phosphate metabolism (Fang et al. 2014; Ford et al. 2011). Current treatment strategies mainly focus on controlling mineral metabolism and slowing disease progression, including phosphate binders, active vitamin D supplementation, and medications like metformin (Neyra and Chawla 2021; Yanai et al. 2021; Baigent et al. 2022). However, these treatments still have limitations in improving patient outcomes and reducing complications, particularly regarding the long-term effects on bone metabolism. Some suggest that targeting the cross-linking between osteoclasts and osteoblasts may have therapeutic effects on CKD-MBD (Hung et al. 2020), providing a direction for new treatment strategies. Osteoclasts are bone-resorbing cells responsible for releasing calcium and phosphate from the bone matrix into circulation, whereas osteoblasts are bone-forming cells regulating bone remodeling (Clézardin et al. 2021). In CKD, the uncoupling of interactions between osteoclasts and osteoblasts disrupts bone balance, contributing to the pathogenesis of MBDs (Iseri et al. 2020). Therefore, seeking new targets to restore osteoblast differentiation is important in managing CKD-MBD (An et al. 2016; Zhu et al. 2021).

Casein kinase 2 (CK2) is a kinase that plays a vital role in regulating bone metabolism, with its expression and activity significantly altered in CKD (Bouhaddou et al. 2020). It is a multifunctional kinase that phosphorylates substrates in multiple cellular lineages of eukaryotes (Hitz et al. 2021). Recent research indicates that CK2 regulates the activity of osteoclasts and osteoblasts, both crucial cell types for bone remodeling and balance (Kim et al. 2022a, b). Targeting CK2 activity in CKD-induced MBD may offer a promising strategy to balance bone metabolism, improve BMD, and prevent bone loss and fractures (Pazianas and Miller 2021). The peptide CK2.3 acts downstream of bone morphogenetic protein receptor type Ia (BMPRIa), inducing osteogenesis in vivo and in vitro (Vrathasha et al. 2018). As reported recently, CK2.3 can enhance Bone Formation in ovariectomized rats (Sequeira et al. 2020). Notably, a bioinformatics prediction conducted in current studies identified runt-related transcription factor 2 (RUNX2) and ubiquitin-specific protease 7 (USP7) as essential genes for CKD-MBD (Kim et al. 2020). RUNX2 is a transcription factor that controls osteoblast differentiation and is responsible for bone formation, positively impacting bone mineral density (BMD) (Huck et al. 2020). Recent studies suggest that RUNX2 is involved in regulating bone metabolism and disease progression in CKD individuals (Kim et al. 2020). Interestingly, research has found that CK2 phosphorylates RUNX2 to stabilize its expression by detaching RUNX2 from ubiquitin-dependent proteasomal degradation, thereby emphasizing the importance of the CK2/USP7 deubiquitination pathway for Bone Formation and suggesting potential therapeutic approaches for improper mineralization disorders (Kim et al. 2020).

RUNX2 is a fundamental transcription factor in skeletal development and a key regulator of osteoblast differentiation (Komori 2022). As previously reported, the downregulation of RUNX2 by ActRIIA during the early stages of CKD contributes to the development of osteoporosis components of MBD and cardiovascular diseases (Williams et al. 2018). Earlier studies have found lower protein expression of RUNX2 in rat models of osteoporosis, which may impact BMD (gianni 2020). Interestingly, research has shown that CK2 can phosphorylate a specific isoform of USP7 at serine 18 (Khoronenkova et al. 2012). USP7, also known as herpesvirus-associated ubiquitin-specific protease, is a deubiquitinase (Al-Eidan et al. 2022). Observations indicate that USP7 is downregulated in tissues of osteoporotic mice, and overexpression of USP7 can increase BMD and promote osteoblast differentiation (Lu et al. 2021).

Current treatment strategies for CKD-MBD primarily focus on controlling mineral metabolism and slowing disease progression, yet there are still deficiencies in improving bone metabolism and reducing the risk of fractures. To address these issues, we hypothesized in this study that CK2 might impact the development of CKD-MBD by modulating RUNX2 and USP7, and we conducted in vivo and in vitro experiments in CKD-MBD mice and pre-osteoblasts to validate this assumption. Our research revealed the crucial roles of RUNX2 and USP7 in regulating Bone Formation and metabolism in CKD-MBD, uncovering not only the complex effects of their interactions on Bone metabolism but also providing new potential targets for treating CKD-MBD. By elucidating the interactions among CK2, RUNX2, and USP7, novel therapeutic avenues for bone loss and fractures in CKD patients can be explored, thus addressing the gaps in current treatment approaches.

## Materials and methods

### Bioinformatics analysis

The Gene Expression Omnibus (GEO) database (https://www.ncbi.nlm.nih.gov/gds) was used to download the gene expression microarrays related to CKD: GSE88925 and GSE148084. The GSE88925 chip comprises 10 samples from male mice with CKD and 8 samples from sham-operated mice, sourced from the vascular tissues of musculus soleus. Meanwhile, the GSE148084 chip consists of 12 samples from sham-operated mice and 12 samples from mice with CKD. The differential expression of mRNA was studied using the Limma package (version: 3.40.2) in R software, with the threshold for differential gene expression set at|log2FC|>1 and P-value < 0.05.

For the GSE88925 dataset, the Weighted Gene Co-expression Network Analysis (WGCNA) was performed in R using the “WGCNA” package. The analysis included hierarchical clustering using the Hclust function, selecting an optimal soft threshold β through the “pickSoftThreshold” function, transforming the adjacency matrix, computing the Topological Overlap Matrix (TOM), and constructing a hierarchical clustering dendrogram to assign similar gene expressions into different modules, with a minimum gene number set at 50 per module. A threshold of 0.25 was defined as the cut height for merging potentially similar modules. Subsequently, module eigengenes (MEs) were used to summarize the expression profiles of each module, and the correlation between MEs and traits was calculated. The Jvenn tool was utilized to identify the overlapping genes between co-expression module features and Differentially Expressed Genes (DEGs).

The interactome network of target genes was obtained from the STRING database (https://string-db.org) and imported into Cytoscape software (version 3.8.2) to construct a regulatory network. The network relationships were then analyzed using Cytoscape, with conditional filtering applied to the results, visualized using different colors to represent the degree values (Wu et al. 2022).

### Establishment of CKD-MBD mouse model

Forty-two 8-week-old male BALB/c mice were purchased from Beijing Vital River Laboratory Animal Technology Co., Ltd. (strain code: 211, Beijing, China) and housed in a Specific Pathogen-Free (SPF) animal facility under controlled conditions: humidity at 60–65%, temperature maintained at 22–25 °C, with a 12-hour light-dark cycle. The experimental procedures commenced after a one-week acclimatization period. Approval for the animal experiments was obtained from the Institutional Animal Care and Use Committee.

The 5/6 nephrectomy was performed under isoflurane anesthesia. For the model group, the left kidney was exposed through a left incision and decapsulated to prevent injury to the ureter and adrenal gland. Subsequently, the upper and lower poles of the kidney were excised, and hemostasis was achieved using a microfibrillar collagen hemostat (Gelaspon, Chauvin Ankerpharm, Berlin). The removed poles were weighed to confirm a 2/3 nephrectomy. One week later, the entire right kidney was removed through a right incision.

In the Sham control group, only the left kidney was exposed through a left incision, and the muscle and skin were repositioned and sutured appropriately after each flank incision. Post-surgery, the operated mice were fed a high-phosphate diet containing 0.9% phosphate and 0.6% calcium, while Sham group mice received a regular diet. After 12 weeks of feeding, the mice were euthanized, and blood serum and bone tissues were collected for further experiments.

Thirty mice were randomly divided into 7 groups (6 mice per group): Sham group, CKD-MBD group, Sham + sh-NC group, sh-NC group (CKD-MBD mice injected with sh-NC lentivirus), sh-RUNX2 group (CKD-MBD mice injected with sh-RUNX2 lentivirus), oe-NC + sh-NC group (CKD-MBD mice injected with oe-NC + sh-NC lentivirus), oe-CK2 + sh-NC group (CKD-MBD mice injected with oe-CK2 + sh-NC lentivirus), oe-CK2 + sh-RUNX2 group (CKD-MBD mice injected with oe-CK2 + sh-RUNX2 lentivirus). The lentiviral gene overexpression vector LV5-GFP and the gene silencing vector pSIH1-H1-copGFP were template vectors to construct the lentivirus packaging system. The sh-NC, sh-RUNX2, and sh-USP7 lentiviruses were obtained from Shanghai Gemma Pharmaceutical Technology Co., Ltd. Three days before modeling, each lentivirus was injected intravenously via the tail vein, and once a week for 6 consecutive weeks after modeling, with a dose of 1 × 10^8^TU/200 µl. The lentiviral transfection sequences are listed in Table S1 (Zaloszyc et al. 2023).

### Observation of bone tissue morphology

Calcium chromate green (C2012, Shanghai Biyun Tian Biological Technology Co., Ltd., Shanghai, China) at a dose of 25 mg/kg and alizarin-3-methylimino diacetic acid (Sigma, A3882, Sigma, USA) at a dose of 50 mg/kg were injected into mice 5 days before euthanasia, dissolved in a 2% sodium bicarbonate solution. After fixation in 10% neutral buffered formalin for 2 days, the undecalcified femoral samples were embedded in methyl methacrylate. Longitudinal Sect. (5 μm thick) were prepared at the proximal and distal ends and stained with the McNeal trichrome method for bone-like assessment, toluidine blue for osteoblasts, and tartrate-resistant acid phosphatase (TRAP) for osteoclasts. An area of interest was defined, and the erosion perimeter/bone perimeter (E.Pm/B.Pm), number of osteoclasts (Oc.N/BL), osteoclast perimeter/bone perimeter (Oc.Pm/B.Pm), and Bone Formation Rate (BFR/Ob) per osteoblast were measured using a Nikon Optiphot 2 microscope connected to a semi-automatic analysis system. Measurements were taken on two slices/samples (approximately 25 μm apart) following ASBMR standards and summed before standardization to obtain a single measurement/sample (Mohamed et al. 2023).

### Serum biochemical analysis

Blood was collected post-mortem from the eyeball of mice, left to stand, and then centrifuged at 1500 rpm at 4 °C for 20 min to obtain serum. The levels of parathyroid hormone (PTH) (ml002044; Shanghai Enzyme-linked Biotechnology Co., Ltd., Shanghai, China), alkaline phosphatase (ALP) (ml002044; Shanghai Enzyme-linked Biotechnology Co., Ltd., Shanghai, China), and fibroblast growth factor 23 (FGF23) (60-6300; Immunotopics, San Clemente, CA, USA) were determined using ELISA assay kits. Additionally, serum markers of bone turnover, C-terminal telopeptide of type I collagen (CTX-I) (ml002251; Shanghai Enzyme-linked Biotechnology Co., Ltd., Shanghai, China), and procollagen type 1 N-terminal propeptide (P1NP) (ml063063; Shanghai Enzyme-linked Biotechnology Co., Ltd., Shanghai, China), were also measured using ELISA assay kits (Heveran et al., 2016).

### Micro-CT 3D scan

Micro-CT 3D scanning was employed for the qualitative and quantitative assessment of trabecular and cortical bone microstructure, conducted by researchers blinded to the genotype of the analyzed animals. The femurs extracted from mice were scanned using a MicroCT 35 scanner (Scanco Medical, Switzerland) with a spatial resolution of 7 μm. For trabecular bone analysis at the distal femur, the upper 2.1 mm region starting from 280 μm proximal to the growth plate was scanned. For cortical bone analysis of the femur and tibia, a 0.6 mm central region was scanned. 3D reconstruction images were obtained from binarized 2D images using distance transformation based on the contouring method (Shen et al. 2023).

### H&E staining

The tibia bones were embedded in paraffin and 4 mm thick tissue sections were prepared for H&E staining. Paraffin sections underwent routine gradient alcohol dehydration, xylene cleaning, deionized water rinsing, and staining with hematoxylin and eosin (C0107, Biyun Tian) for 8 min, followed by rinsing. Differentiation with 1% hydrochloric acid ethanol for 20 s, rebluing with 1% ammonium hydroxide for 30 s, and deionized water rinsing were performed. The sections were counterstained with eosin solution (C0109, Biyun Tian) for 5 min, rinsed in deionized water for 1 min, dehydrated, dried, and sealed. Morphological structural changes in skin tissues were observed under an optical microscope, followed by the analysis of bone volume fraction (BV/TV) in stained sections using ImageJ software (NIH, Bethesda, MD, USA) (Martín-Vírgala et al. 2023).

### Cell culture and group transfection

Murine Osteogenic precursor cell lines MC3T3-E1 (GDC0188, obtained from the Chinese Academy of Typical Culture Collection, Wuhan, China) and HEK293T cell lines (GDC0187, obtained from the Chinese Academy of Typical Culture Collection, Wuhan, China) were selected for in vitro cell experiments to investigate the impact of relevant factors on the proliferation and differentiation of Osteogenic precursor cells MC3T3E1. Both cell lines MC3T3-E1 and HEK293T were cultured following the instructions provided for the cell lines.

Overexpression and silencing lentiviruses were purchased from Shanghai Gemma Pharmaceutical Technology Co., Ltd. Logarithmic growth phase MC3T3-E1 cells (4 × 10^5^ per well) were seeded in 6-well cell culture plates. After 24 h of incubation in a cell culture incubator, the respective lentiviruses were used to infect MC3T3-E1 cells in each group. After 48 h of infection, the GFP expression efficiency was observed using a fluorescent microscope. The culture medium was then replaced with a medium containing 4 µg/mL puromycin, and the MC3T3-E1 cells were cultured for at least 14 days. Puromycin-resistant cells were expanded in medium containing 2 µg/mL puromycin for 9 days, followed by transfer to puromycin-free medium to obtain stably overexpressed or silenced MC3T3-E1 cells. The transfection groups were oe-NC, oe-RUNX2, oe-USP7, oe-CK2, sh-NC, sh-RUNX2, sh-USP7, and sh-CK2. The silencing lentivirus transfection and sequences can be referred to in Table S1. Three different silencing sequences were constructed for different genes (RUNX2, USP7, and CK2), and the efficiency of silencing for each sequence was validated using reverse transcription quantitative polymerase chain reaction (RT-qPCR) to select the optimal sequence (Figure S1) (Kim et al. 2020; Candellier et al. 2023).

### CCK-8 assay

Cell proliferation experiment results were statistically analyzed using a CCK-8 assay kit (Cat. No: CA1210, Beijing Solarbio Science & Technology Co., Ltd., Beijing, China). Logarithmic growth phase cells were seeded at 1 × 10^4^ cells per well in a 96-well plate and pre-cultured for 24 h. Subsequently, cells were transfected according to groupings, and after 48 h of transfection, 10 µL of CCK-8 reagent was added at 0 h, 24 h, 48 h, and 72 h post-transfection, followed by incubation at 37 °C for 3 h. Each well’s absorbance values at 450 nm wavelength were measured on an ELISA reader. The absorbance values were directly proportional to the cell proliferation in the culture medium, facilitating the generation of cell growth curves (Hsu et al. 2022).

### Immunoprecipitation experiments

To investigate the interaction between RUNX2 and CK2, mouse MC3T3-E1 cells were lysed in lysis buffer containing 50 mM Tris-HCl (pH 7.4), 150 mM NaCl, 1% Triton X-100, 1 mM EDTA, 1 mM EGTA, 50 mM NaF, 1 mM Na3VO4, 1 mM PMSF, and a protease inhibitor cocktail (PPC1010, Sigma, USA) (Kim et al. 2020). The cell lysates were incubated with either IgG control antibody (ab133470; Abcam, Cambridge, UK) or anti-RUNX2 antibody (1:100) (#12556; Cell Signaling, USA)/CK2 antibody (1:100) (XY-KT-1251; Shanghai XuanYa Biotechnology Co., Ltd., Shanghai, China), followed by immunoprecipitation using protein G-conjugated agarose. To investigate the interaction between RUNX2 and USP7, Flag-RUNX2 and HA-USP7 were transfected into HEK293T cells using Effectene transfection reagent (Qiagen). After 48 h, cell lysates were prepared in lysis buffer. Proteins from the cell lysates were immunoprecipitated using Flag antibody (1:100) (A2220; Sigma, USA) or HA antibody (1:100) (sc-7392 AC; Santa Cruz, USA), followed by SDS-PAGE, transferred onto Immobilon-P membrane (ISEQ07850; Millipore, USA), and immunoblotting with specific antibodies (Dai et al. 2020).

### Deubiquitination experiments

To assess the ability of HAUSP to deubiquitinate RUNX2, plasmids expressing Flag-RUNX2 and His-ubiquitin were co-transfected into MC3T3-E1 cells under conditions where HAUSP expression plasmid was absent or present at different concentrations. After 48 h, cells were treated with 10 µM MG132 (474790; Millipore, USA) for 6 h, lysed in denaturing buffer (8 M urea, 50 mM Tris pH 8.0, 1.0 mM Tris pH 8.0, 1.00% imidazole, 10 mM β-mercaptoethanol), and immunoprecipitated using Ni-NTA beads. Immunoprecipitated materials were subjected to SDS-PAGE and immunoblotted with anti-RUNX2 antibody (#12556; Cell Signaling).

Myc-RUNX2 was co-transfected with sh-NC/sh-CK2 into HEK293T cells. After 48 h, cells were treated with 10 µM MG132 for 6 h, lysed, immunoprecipitated using Myc-conjugated agarose, and immunoblotted with rabbit anti-ubiquitin antibody (1:100) (ZRB2150; Sigma).

Plasmids for Flag/HA-HAUSP were obtained from Bert Vogelstein (#16655) and deposited in Addgene. HA-HAUSP was subcloned from this source. The construct for HA-UBB (ubiquitin) was obtained from Edward Yeh (#18712) and used to generate the plasmid for His-ubiquitin through subcloning (Yang et al. 2022).

### Phosphorylation experiments

Recombinant CK2 (200 ng, P6010; USA) and RUNX2 (300 ng, TP760214, Origene, Beijing, China) were incubated at 30 °C for 15 min in a kinase buffer containing 10 µCi of γ32P-ATP (PerkinElmer) (20 mM HEPES, pH 7.5, 20 mM MgCl2, 1 mM EDTA, 2 mM NaF, glycerol phosphate, 1 mM DTT, 10 µM ATP). Phosphorylated proteins were visualized by autoradiography (Hannigan et al. 2011).

### **RUNX2** protein half-life experiment

Cells were treated with cycloheximide (CHX) (100 µg/mL, No. C7698, Sigma-Aldrich) for 0, 2, 4, 6, and 8 h. Cell proteins were collected for immunoblot analysis to determine the half-life of RUNX2 (Song et al. 2023).

### RT-qPCR

Cellular total RNA was extracted using Trizol (16096020, Thermo Fisher Scientific, USA) and reverse transcribed into cDNA using the PrimeScript RT Kit (Takara Biotechnology Ltd., Dalian, China) and the PrimeScript miRNA RT Kit (Takara Biotechnology Ltd., Dalian, China). RT-qPCR experiments followed the manufacturer’s instructions and were conducted with the RT-qPCR Kit (Q511-02, Vazyme Biotech, Nanjing). The reaction mixture included 2 µL of cDNA template, 0.2 µL each of forward and reverse primers, and 10 µL of RT-qPCR Mix, made up to a total volume of 20 µL with RNAase-free water. PCR amplification was performed in a Bio-Rad real-time quantitative PCR instrument CFX96 under the following conditions: initial denaturation at 95 °C for 30 s, denaturation at 95 °C for 10 s, annealing at 60 °C for 30 s, extension at 72 °C for 30 s, with a total of 40 cycles, and a melting curve from 60 to 95 °C. GAPDH was used as an internal reference. Primer sequences were designed and provided by Shanghai Sangon Biotech (Shanghai, China), as listed in Table S2. Each RT-qPCR was set up in triplicate and repeated three times. The 2^−ΔΔCt^ method was used to determine the fold change in target gene expression between the experimental and control groups, calculated using the formula: ΔΔCT = ΔCt experimental group - ΔCt control group, where ΔCt = Ct target gene - Ct internal reference gene. Ct represents the cycle threshold at which the fluorescence signal reaches a predefined threshold, indicating exponential amplification (Kim et al. 2020).

### Western blot analysis

Tissues and cells from different group (bone protein was extracted from femoral cortical bone) were collected and individually lysed in ice-cold RIPA lysis buffer (P0013B, Biyuntian, Shanghai, China) containing 1% PMSF for 30 min at 4 °C with centrifugation at 14,000 rpm, followed by collection of the supernatant. Nuclear proteins were extracted using a subcellular fractionation kit (AR0106, Boside, China). The protein concentration of the samples was determined by a BCA assay (AR0197, Boside, China). Subsequently, 50 µg of protein was denatured by boiling in a 5× loading buffer at 100 °C for 10 min. Proteins were separated by SDS-PAGE based on their molecular weights using separating and stacking gels, and then transferred onto a PVDF membrane (ISEQ07850; Millipore, USA) by wet transfer. The membrane was blocked with 5% BSA at room temperature for 1 h and then incubated overnight at 4 °C with primary antibodies against rabbit polyclonal antibodies for RUNX2 (ab236639; Abcam, Cambridge, UK), CK2 (10992-1-AP; Proteintech, USA), USP7 (ab108931; Abcam, Cambridge, UK), and GAPDH (ab108931; Abcam, Cambridge, UK), with β-Actin used as an internal control. After washing, the membrane was further incubated with an HRP-conjugated secondary antibody IgG (ab6721, 1:5000, Abcam, UK) for 2 h. The membrane was then washed three times with TBST for 5 min each, then detected using a chemiluminescence imaging system (Chiu et al. 2020).

### Statistical analysis

The research data was analyzed using Graphpad Prism. Continuous data were presented as mean ± standard deviation. The independent samples t-test was used for comparisons between two groups, while one-way analysis of variance (ANOVA) followed by Tukey’s post hoc test was used for comparisons among multiple groups. Repeated measures ANOVA was employed with Tukey’s post hoc test to compare data at different time points within groups. A significance level of *P* < 0.05 was considered statistically significant.

## Results

### Downregulation of RUNX2 and USP7 in CKD mice

**Fig. 1:**
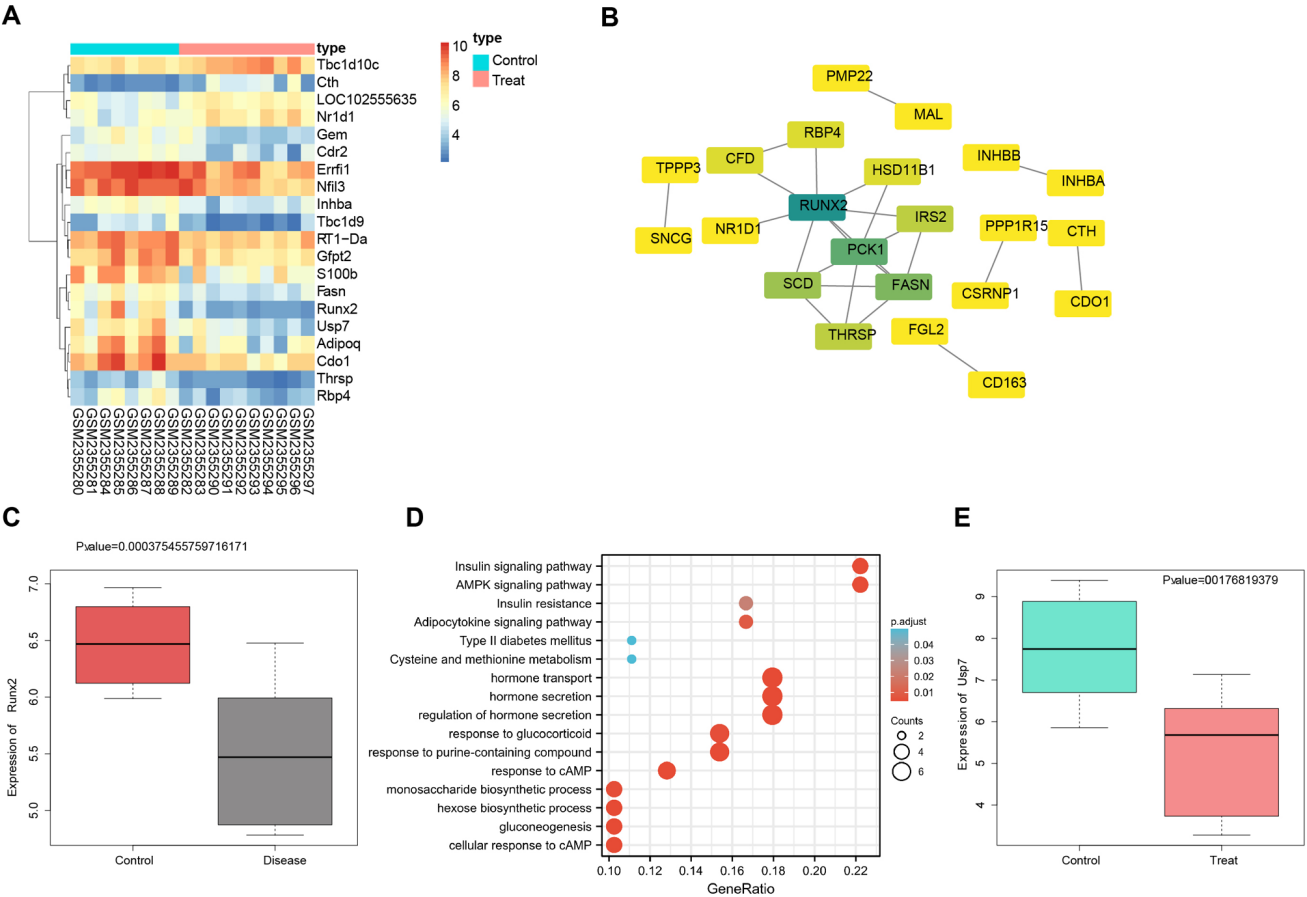
Bioinformatics Analysis to Identify Key Genes Involved in CKD Development. Note: (**A**) Heatmap of DEGs, where the color scale from blue to orange represents expression values from low to high; (**B**) PPI network graph of candidate genes, where the color gradient from green to yellow represents Degree values from high to low; (**C**) Significant downregulation of Runx2 in CKD (Treat, *n* = 10) compared to control samples (Control, *n* = 8); (**D**) Bubble plot of GO and KEGG pathway enrichment analysis for candidate genes, where the size of the circles represents the number of selected genes and the color indicates the significance level of the enrichment analysis; (**E**) Significant downregulation of Usp7 in CKD (Treat, *n* = 12) compared to control samples (Control, *n* = 12)

To identify CKD-associated functional genes, we conducted a differential analysis of CKD-related mRNA expression profiles from the GEO dataset, resulting in 65 DEGs. The top 20 DEGs with the lowest p-values were illustrated in a heatmap (Fig. 1A). Furthermore, utilizing the GEO dataset GSE88925, we performed WGCNA analysis to retain the top 25% of genes with the highest variance for subsequent analysis. Hierarchical clustering of 18 samples was conducted (Figure S2A), using a soft threshold of β = 17 to construct a scale-free network (Figure S2B).

Five co-expression modules were identified in the GSE88925 expression profile, with each color representing a distinct module (Figure S2C). Correlation analysis of module characteristics revealed a significant positive correlation between the co-expression module MEred and CKD, which comprised a total of 176 genes (Figure S2D). Through the jvenn tool, the intersection of feature genes in the co-expression module MEred and DEGs yielded 43 candidate genes (Figure S2E). PPI analysis of the 43 candidate genes indicated that Runx2 occupied a central position in the network with the highest Degree (Fig. 1B). Differential analysis demonstrated that Runx2 was significantly downregulated in CKD samples (Fig. 1C). GO and KEGG pathway enrichment analysis of the candidate genes revealed their involvement in various signaling pathways, including insulin and AMPK signaling, affecting the progression of CKD (Fig. 1D).

Existing literature suggests that Runx2 plays a central role in insulin-regulated signal transduction and execution (Adhami et al. 2011). CK2 phosphorylates RUNX2, recruiting the deubiquitinase herpesvirus-associated USP7, thus stabilizing RUNX2 by shifting it from ubiquitin-dependent proteasomal degradation (Kim et al. 2020). Differential analysis based on the CDK-related chip dataset GSE148084 revealed significant downregulation of the deubiquitinase Usp7 in CKD samples (Fig. 1E). We hypothesize that Runx2 may influence CKD through Usp7.

### Silencing RUNX2 prevents proliferation and differentiation of osteogenic precursor cells

To further investigate the potential molecular mechanisms by which RUNX2 influences bone formation and bone metabolism, we conducted in vitro cell experiments using osteogenic precursor cells MC3T3-E1. The RT-qPCR results (Figure S3A) showed that the expression of RUNX2 in MC3T3-E1 cells significantly increased in the oe-RUNX2 group compared to the oe-NC group and notably decreased in the sh-RUNX2 group compared to the sh-NC group, while RUNX2-I remained largely unaffected. The CCK-8 experiment results (Figure S3B) indicated a significant increase in the number of MC3T3-E1 cells in the oe-RUNX2 group compared to the oe-NC group; however, a significant decrease was observed in the sh-RUNX2 group compared to the sh-NC group.

Further RT-qPCR analysis was performed to evaluate the expression of hallmark genes (ALP, Collagen-1, and Osteocalcin) involved in osteogenic differentiation. The results (Figure S3C) revealed a significant decrease in the expression of ALP, Collagen-1, and Osteocalcin in MC3T3-E1 cells of the sh-RUNX2 group compared to the sh-NC group, while a significant increase was observed in ALP, Collagen-1, and Osteocalcin expression in the oe-RUNX2 group compared to the oe-NC group.

These findings demonstrate that silencing RUNX2 prevents the proliferation and differentiation of osteogenic precursor cells, whereas overexpression of RUNX2 promotes the differentiation of osteogenic precursor cells into mature osteoblasts.

### USP7 stabilizes RUNX2 expression through deubiquitination

**Fig. 2:**
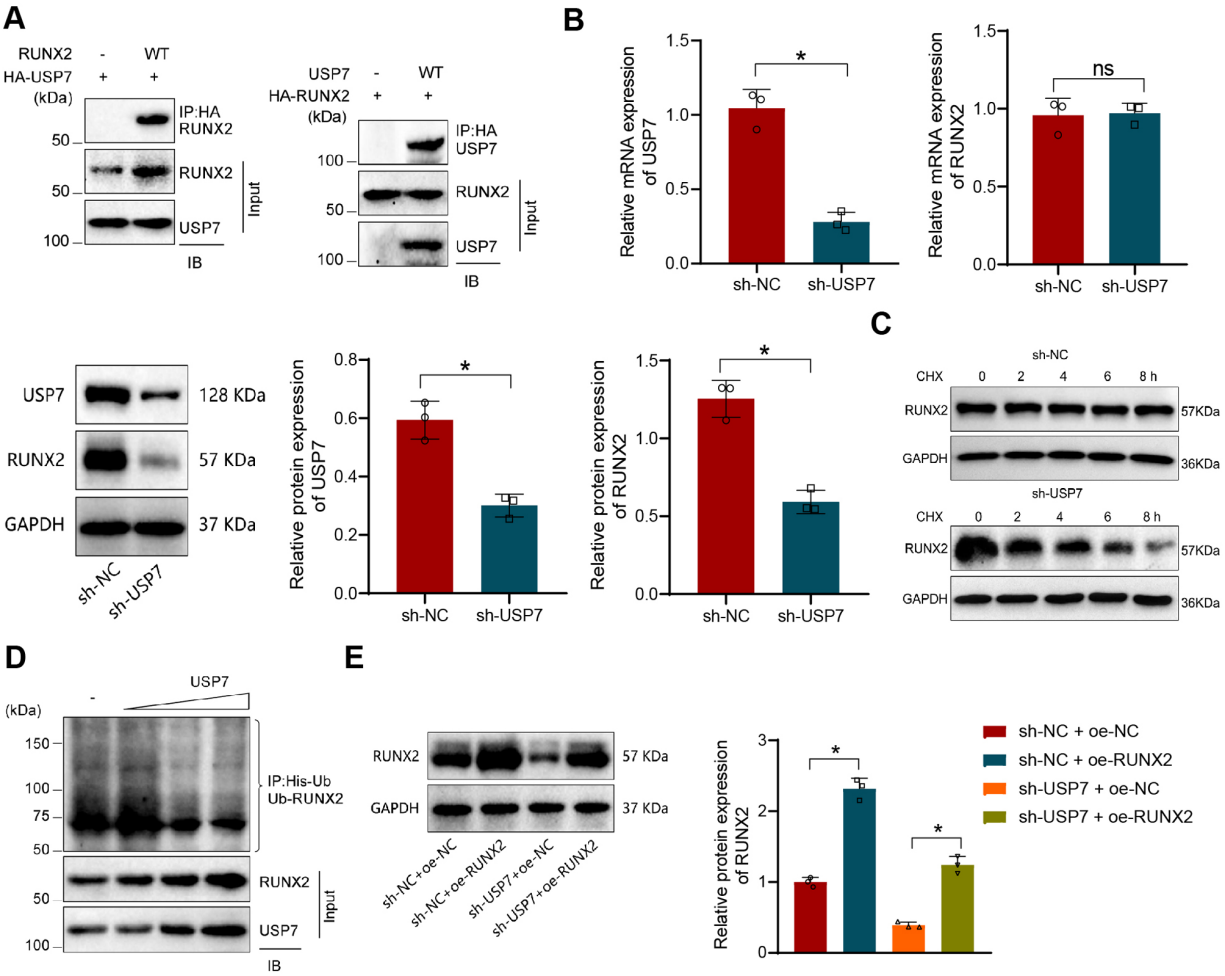
In vitro Cell Experimental Validation of the Interaction between USP7 and RUNX2. Note: (**A**) Immunoprecipitation assay of USP7 binding with RUNX2; (**B**) RT-qPCR and Western Blot analysis of mRNA and protein levels of USP7 and RUNX2 in HEK293T cells in sh-NC and sh-USP7 groups; (**C**) Evaluation of the protein half-life of RUNX2; (**D**) In vitro deubiquitination experiment measuring the impact of USP2 on the ubiquitination levels of RUNX2; (**E**) Western Blot analysis of the protein levels of RUNX2 in HEK293T cells in different groups, where “ns” indicates no significant difference between the groups (*P* > 0.05), and * denotes a significant difference between the groups (*P* < 0.05); all cellular experiments were performed in triplicate

Previous studies have indicated that USP7 regulates osteogenic differentiation by stabilizing RUNX2 (Kim et al. 2020). To begin with, we verified the interaction between USP7 and RUNX2 through co-immunoprecipitation experiments (Fig. 2A). Results from RT-qPCR and Western Blot assays revealed that knockdown of USP7 led to a reduction in the protein expression of RUNX2 without affecting its mRNA levels (Fig. 2B). The half-life of the RUNX2 protein was analyzed using CHX tracing to ascertain the impact of USP7 on the stability of RUNX2. The findings demonstrated a shortened half-life of the RUNX2 protein following USP7 knockdown (Fig. 2C). Ubiquitination assays conducted in vitro indicated a substantial decrease in the ubiquitination levels of RUNX2 and a consequent increase in the protein levels of RUNX2 with elevated USP7 levels (Fig. 2D). Furthermore, Western Blot results showed a significant increase in the protein content of RUNX2 in the sh-NC + oe-RUNX2 group compared to the sh-NC + oe-NC group. Similarly, the sh-USP7 + oe-RUNX2 group exhibited a significant increase in RUNX2 protein content compared to the sh-USP7 + oe-NC group (Fig. 2E).

These results collectively suggest that USP7 stabilizes RUNX2 expression through deubiquitination.

### CK2 phosphorylates and activates RUNX2 to recruit USP7, thereby stabilizing RUNX2 expression

**Fig. 3:**
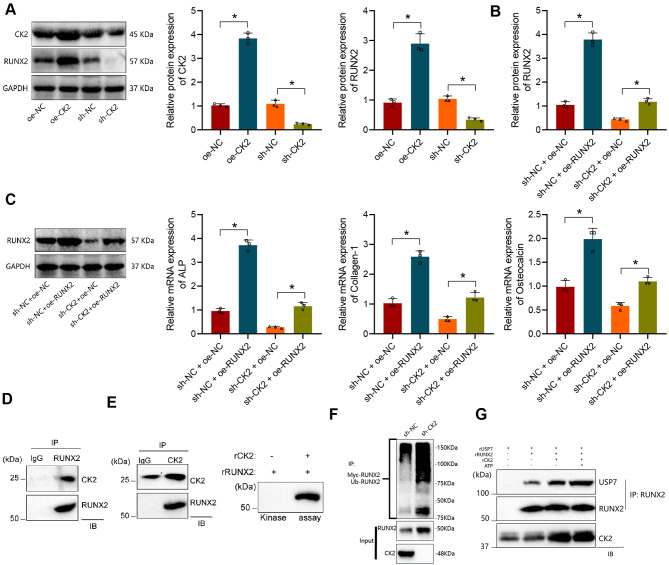
In vitro Cell Experimental Validation of CK2 and RUNX2/USP7 Interaction. Note: (**A**) Western Blot analysis of the protein expression levels of CK2 and RUNX2 in various groups of MC3T3-E1 cells; (**B**) Western Blot analysis of the protein expression levels of RUNX2 in different groups of MC3T3-E1 cells; (**C**) RT-qPCR analysis of the relative expression levels of osteoblast marker genes (ALP, Collagen-1, and Osteocalcin) in different groups of MC3T3-E1 cells; (**D**) Immunoprecipitation experiment to detect the interaction between RUNX2 and CK2; (**E**) Phosphorylation kinase assay assessing the regulatory role of CK2 in the phosphorylation of RUNX2; (**F**) Ubiquitination experiment measuring the ubiquitination levels of RUNX2 in various groups of cells after treatment with MG132; (**G**) In vitro cell experimental validation of the mutual regulation among CK2, USP7, and RUNX2. * indicates a significant difference between the groups (*P* < 0.05), and all cellular experiments were conducted in triplicate

Previous research has indicated that CK2-induced phosphorylation of RUNX2 recruits USP7, leading to the deubiquitination and stabilization of RUNX2 expression in osteoblasts (Kim et al. 2020). In this study, we transfected MC3T3-E1 cells with oe-NC/oe-CK2 or sh-NC/sh-CK2. Western Blot analysis revealed a significant upregulation of CK2 and RUNX2 expression levels in cells of the oe-CK2 group compared to oe-NC, and a significant decrease in cells of the sh-CK2 group compared to sh-NC (Fig. 3A).

Subsequently, we further transfected sh-NC/shCK2 cells with oe-NC/oe-RUNX2 to obtain sh-NC + oe-NC/sh-NC + oe-RUNX2 cells and sh-CK2 + oe-NC/sh-CK2 + oe-RUNX2 cells. Western Blot results demonstrated a significant increase in the protein content of RUNX2 in cells of the sh-NC + oe-RUNX2 group compared to the sh-NC + oe-NC group and also in cells of the sh-CK2 + oe-RUNX2 group compared to sh-CK2 + oe-NC group (Fig. 3B). Subsequently, the expression levels of osteoblast marker genes (ALP, Collagen-1, and Osteocalcin) were evaluated by RT-qPCR. The results showed a significant elevation in the expression of osteoblast marker genes in the sh-NC + oe-RUNX2 group compared to sh-NC + oe-NC group, while in the sh-CK2 + oe-RUNX2 group, a trend of increased expression of osteoblast marker genes was observed compared to sh-CK2 + oe-NC group, indicating the essential role of RUNX2 in mediating osteoblastic cell differentiation (Fig. 3C).

To investigate the regulatory role of CK2 on RUNX2, we conducted co-immunoprecipitation analysis in HEK293T cells, demonstrating the binding of CK2 to RUNX2 (Fig. 3D). Moreover, in vitro kinase assays using recombinant CK2 (rCK2) and RUNX2 (rRUNX2) revealed that CK2 can activate RUNX2 through phosphorylation modifications (Fig. 3E).

Furthermore, knockdown of CK2 resulted in decreased protein expression of RUNX2 in HEK293T cells. However, treatment with the proteasome inhibitor MG132 significantly increased the ubiquitination levels of RUNX2 (Fig. 3F). In an in vitro reaction system, adding rCK2 and ATP enhanced the interaction between rRUNX2 and rUSP7 (Fig. 3G).

In conclusion, the above results indicate that CK2 phosphorylates and activates RUNX2 to recruit USP7, thereby stabilizing RUNX2 expression, and promoting the differentiation of osteogenic precursor cells into mature osteoblasts.

### Decreased bone formation and disrupted bone metabolism in CKD-MBD mice

Recent studies have confirmed (2022 J Clin Invest article) that, in both in vivo and in vitro CKD models, RUNX2 promotes osteoblast differentiation (Li et al. 2022) and also inhibits osteoclast generation (Xin et al. 2020). Therefore, we hypothesize that RUNX2 may be closely associated with CKD-MBD development.

**Fig. 4:**
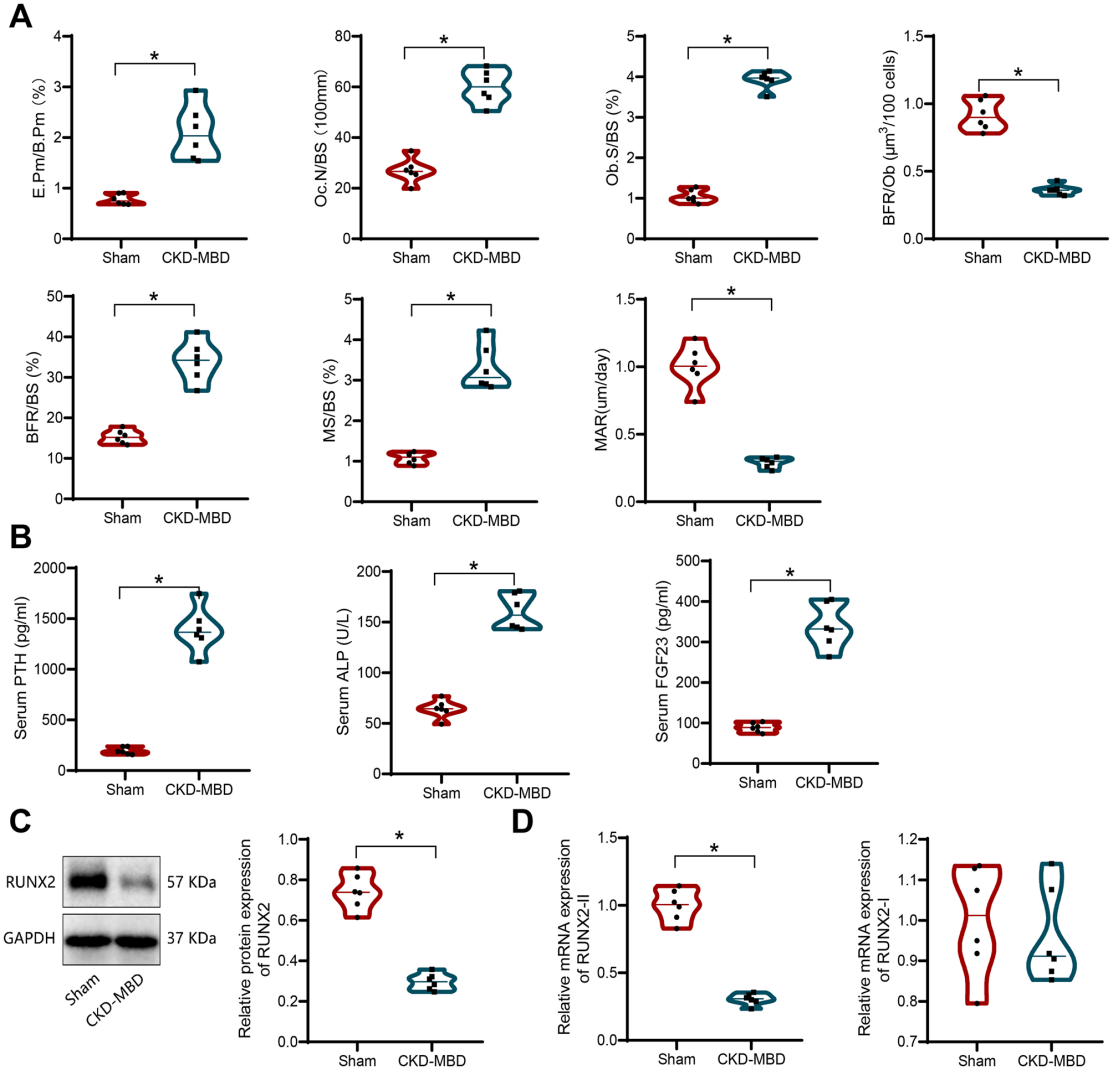
Bone Formation and Bone Metabolism State in CKD-MBD Mice. Note: (**A**) Histomorphometric analysis of bone tissue in the two groups of mice, including erosion perimeter/bone perimeter (E.Pm/B.Pm), osteoclast number (Oc.N/BS), osteoclast perimeter/bone perimeter (Obs/BS), and Bone Formation rate (BFR/Ob), BFR/BS, MS/BS, and MAR; (**B**) ELISA measurement of serum PTH, ALP, and FGF23 levels in the two groups of mice; (**C**) Western blot analysis of the expression of RUNX2 protein in bone tissue of the two groups of mice; (**D**) RT-qPCR measurement of RUNX2 RNA expression levels. * indicates comparisons between the two groups, where *P < 0.05 denotes significance. Each group consisted of 6 mice

Using a CKD-MBD mouse model, we conducted bone histomorphometric analyses on Sham and CKD-MBD groups. The results showed that the CKD-MBD group exhibited significantly increased eroded perimeter per bone perimeter (E.Pm/B.Pm), osteoclast number per bone surface (Oc.N/BS), and osteoblast surface per bone surface (Ob.S/BS) compared to the Sham group. However, due to delayed mineralization, the bone formation rate per osteoblast surface (BFR/Ob) was reduced in the CKD-MBD group (Fig. 4A).

Additionally, ELISA results demonstrated that serum levels of PTH, FGF23, and ALP were significantly elevated in the CKD-MBD group compared to the Sham group (Fig. 4B). Western blot analysis of bone tissue revealed that RUNX2 protein expression was significantly decreased in the CKD-MBD group compared to the Sham group (Fig. 4C), and this decrease was primarily attributed to reduced expression of the RUNX2-II isoform (Fig. 4D).

### Silencing of RUNX2 led to a decrease in bone density and strength in CKD-MBD mice

**Fig. 5:**
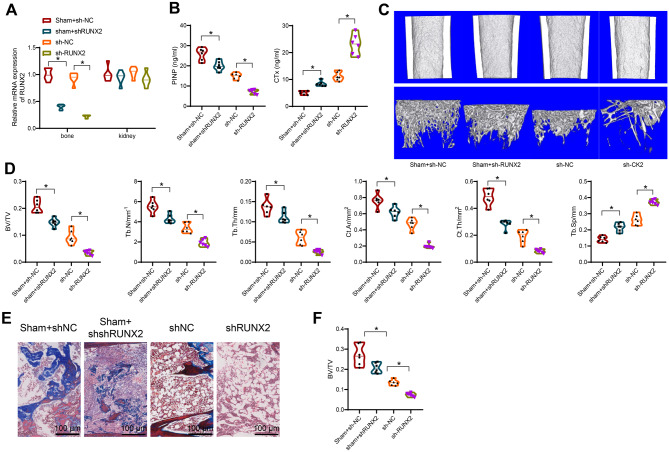
Impact of RUNX2 Silencing on Bone Turnover Rate, Bone Density, Bone Strength, and Osteoclastogenesis in CKD-MBD Mice. Note: (A) RT-qPCR to detect RUNX2 expression levels in various tissues; (**B**) ELISA assessment of expression levels of PINP and CTx in the serum of each group of mice; (**C**) Micro-CT 3D scanning (200 μm) of trabecular and cortical bone regions in each group of mice; (**D**) Quantitative evaluation of bone parameters from the Micro-CT 3D scanning results, including BV/TV, Tb. N (mm^− 1^), Tb. Th (mm), Tb. Sp, mm, Ct. Ar (mm^2^), and Ct. Th (mm^2^) (* indicates comparison with sh-NC group, *P* < 0.05); (E) H&E staining of the tibial bone tissue in each group of mice (200 μm); (F) Quantitative histomorphological evaluation of the MASSON stained tissues. * denotes significance in comparisons between the two groups (*P* < 0.05). Each group comprised 6 mice

To further investigate the impact of RUNX2 on bone metabolism in CKD-MBD mice, we conducted lentiviral silencing of RUNX2 in these mice. We also measured RUNX2 expression in non-bone tissues such as the kidneys. We found that in non-bone tissues, the expression of RUNX2 remained relatively unchanged after silencing (Fig. 5A). As shown in Fig. 5B, compared to the sham + sh-NC group, the sh-NC group mice exhibited a significant decrease in serum PINP content and increased CTx expression. Furthermore, compared to the sh-NC group, the sh-RUNX2 group mice showed further decreases in serum PINP content and increased CTx expression, indicating an enhanced bone turnover rate in CKD-MBD mice after RUNX2 silencing.

In addition, Micro-CT 3D scanning analysis of tibial trabeculae below the proximal growth plate and cortical bones in the midshaft of femurs and tibias showed that sh-RUNX2 group mice had lower trabecular bone density and thinner tibial cortical bone compared to sh-NC group mice (Fig. 5C). Pathological histomorphometric analysis revealed that compared to sh-NC group mice, sh-RUNX2 group mice exhibited significantly lower bone volume fraction (BV/TV), trabecular number (Tb. N), trabecular thickness (Tb. Th), bone area, cortical thickness (Ct. Th), and increased trabecular separation (Tb. Sp) (Fig. 5D), indicating a significant decrease in bone density and strength in sh-RUNX2 group mice. MASSON staining results showed a marked reduction in the percentage of bone tissue volume in the cortical area (Ct. Ar) of sh-RUNX2 group mice (Fig. 5E-F). Furthermore, we found that compared to the Sham group, silencing RUNX2 in the Sham group also caused a decrease in PINP levels, an increase in CTx content, lower trabecular bone density, thinner tibial cortical bone, and a reduction in various histomorphometric parameters (Fig. 5B-F).

In summary, silencing of RUNX2 results in an increased bone turnover rate, decreased bone density, and reduced bone strength in CKD-MBD mice.

### CK2 improves MBD in CKD-MBD mice by regulating RUNX2 expression

**Fig. 6:**
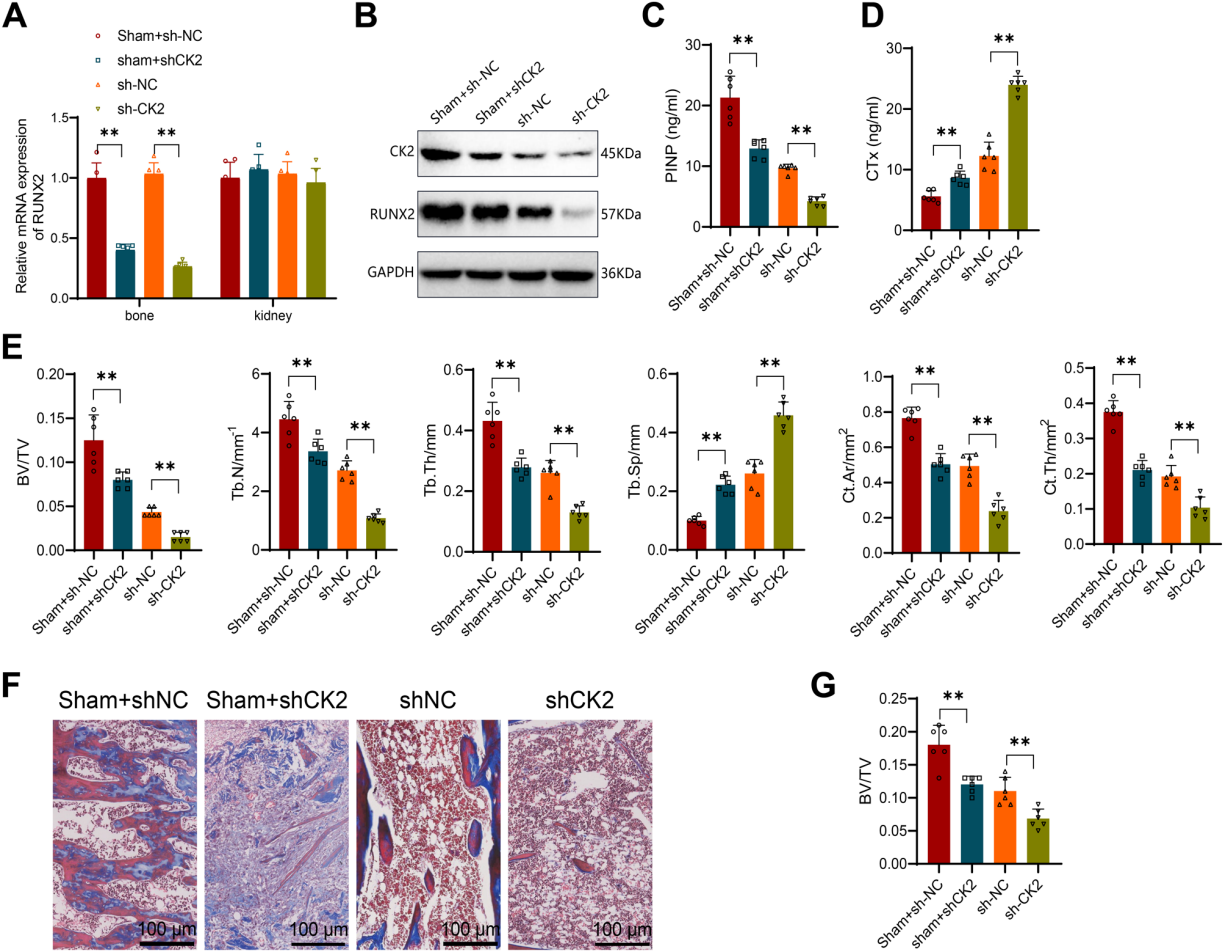
Impact of CK2 Silencing on RUNX2 Expression and Bone Turnover Rate, Bone Density, and Bone Strength in Mice. Note: (**A**) RT-qPCR to detect RUNX2 expression levels in various tissues; (**B**) Western blot analysis of CK2 and RUNX2 protein levels in bone tissue of each group of mice; (**C**) ELISA assessment of PINP and CTx expression levels in the serum of each group of mice; (**D**) Micro-CT 3D scanning (200 μm) of trabecular and cortical bone regions in each group of mice; (**E**) Quantitative evaluation of bone parameters from the Micro-CT 3D scanning results, including BV/TV, Tb. N (mm^− 1^), Tb. Th (mm), Tb. Sp, mm, Ct. Ar (mm^2^), and Ct. Th (mm^2^); (**F**) H&E staining of tibial bone tissue in each group of mice (200 μm); (**G**) Quantitative histomorphological assessment of the MASSON stained tissues. *indicates significance in comparisons between the two groups (*P* < 0.05), and ** denotes significance at *P* < 0.01. Each group consisted of 6 mice

Based on the in vivo mouse experiments confirming that silencing of RUNX2 leads to increased bone turnover rate, decreased bone density, and bone strength in CKD-MBD mice, as well as the partial results of the in vitro experiments showing that CK2 phosphorylates and activates RUNX2, recruiting USP7 to stabilize RUNX2 expression and promote the differentiation of osteogenic precursor cells into mature osteoblasts, we postulated that CK2 could improve MBD in CKD-MBD mice through regulating RUNX2 expression. Initially, we silenced CK2 in CKD-MBD mice via lentivirus. We measured CK2 expression in non-bone tissues such as the kidneys, and found that CK2 expression remained relatively unchanged in these tissues (Fig. 6A).

Western Blot results demonstrated successful silencing of CK2 in mouse bone tissues, accompanied by a downregulation of RUNX2 expression levels (Fig. 6B). Additionally, compared to the sh-NC group, the sh-CK2 group mice exhibited reduced serum PINP content and increased CTx expression (Fig. 6C). Micro-CT 3D scanning analysis of tibial trabeculae below the proximal growth plate and cortical bones in the midshaft of femurs and tibias indicated that the sh-CK2 group mice displayed lower trabecular bone density and thinner tibial cortical bone compared to the sh-NC group (Fig. 6D). Pathological histomorphometric analysis revealed significant reductions in BV/TV, Tb. N, Tb. Th, Ct. Ar, and Ct.Th, as well as an increase in Tb.Sp in sh-CK2 group mice compared to the sh-NC group mice (Fig. 6E). MASSON staining results showed a notable decrease in the proportion of bone tissue volume in the cortical region of sh-CK2 group mice compared to the sh-NC group (Fig. 6F-G). Furthermore, we found that compared to the sham group, silencing CK2 in the sham group also led to a decrease in PINP, an increase in CTx, lower trabecular bone density, thinner tibial cortical bone, and reductions in various histomorphometric parameters (Fig. 6B-G). These results indicate that in the absence of CK2, the expression of RUNX2 is affected, leading to MBD in mice.

**Fig. 7:**
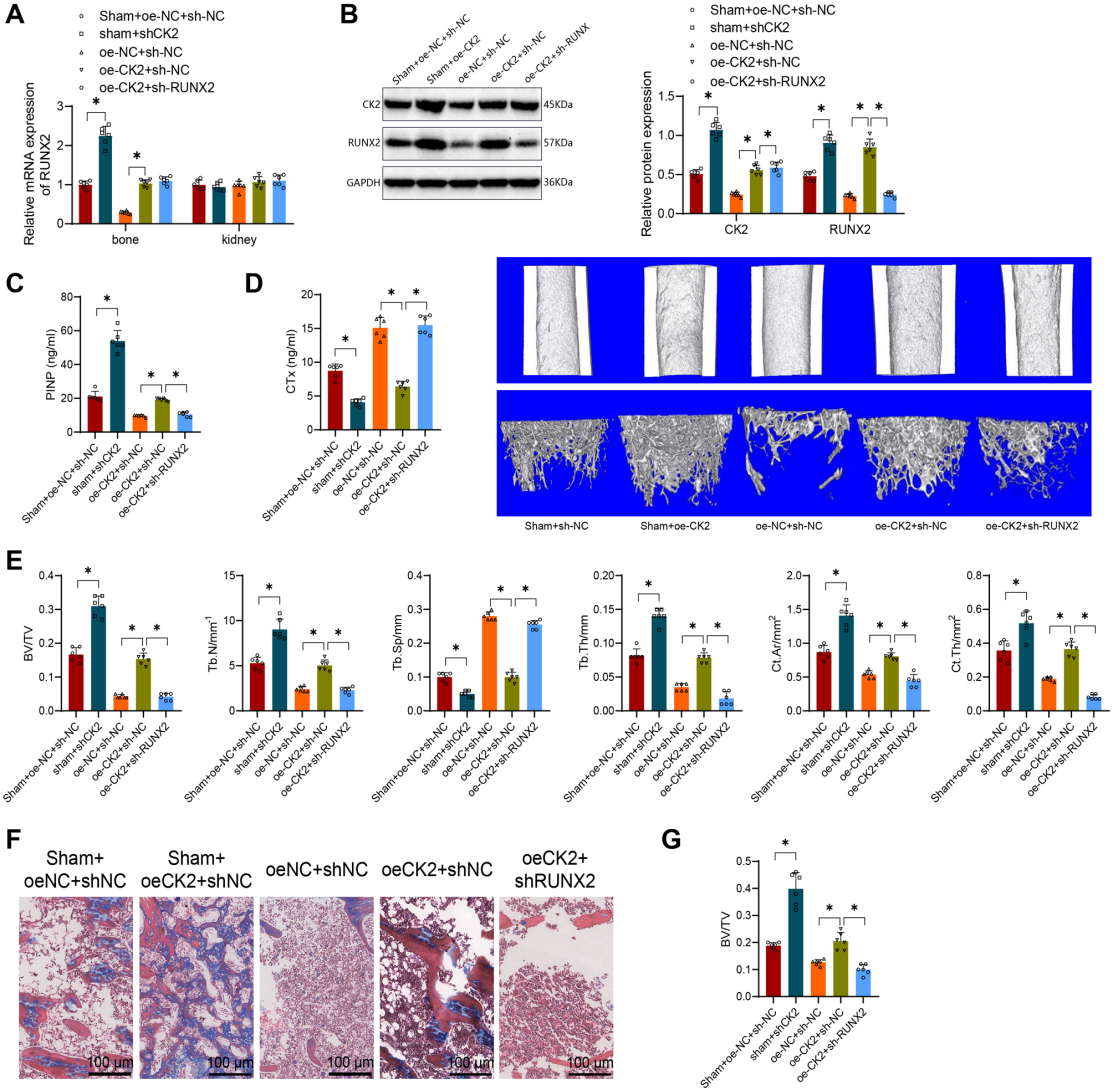
The Impact of CK2 Regulating RUNX2 on Bone Turnover Rate, Bone Density, and Bone Strength in CKD-MBD Mice. Note: (**A**) RT-qPCR to detect RUNX2 expression levels in various tissues; (**B**) Western blot analysis of CK2 and RUNX2 protein levels in bone tissue of each group of mice; (**C**) ELISA assessment of PINP and CTx expression levels in the serum of each group of mice; (**D**) Micro-CT 3D scanning (200 μm) of trabecular and cortical bone regions in each group of mice; (**E**) Quantitative evaluation of bone parameters from the Micro-CT 3D scanning results, including BV/TV, Tb. N (mm^− 1^), Tb. Th (mm), Tb. Sp, mm, Ct. Ar (mm^2^), and Ct. Th (mm^2^); (**F**) H&E staining of tibial bone tissue in each group of mice (200 μm); (**G**) Quantitative histomorphological assessment of the MASSON stained tissues. * indicates significance in comparisons between the two groups (*P* < 0.05). Each group comprised 6 mice.=

To further validate our hypothesis, we conducted lentiviral overexpression of CK2 or simultaneous overexpression of CK2 with silencing of RUNX2 in CKD-MBD mice. In non-bone tissues such as the kidneys, CK2 expression remained relatively unchanged after silencing (Fig. 7A). Western Blot analysis of the protein levels of CK2 and RUNX2 in mouse bone tissues across different groups revealed that, compared to the oe-NC + sh-NC group, the oe-CK2 + sh-NC group exhibited significant increases in CK2 and RUNX2 protein levels; and compared to the oe-CK2 + sh-NC group, the oe-CK2 + sh-RUNX2 group showed no significant difference in CK2 protein levels but a marked decrease in RUNX2 protein levels (Fig. 7B).

**Fig. 8:**
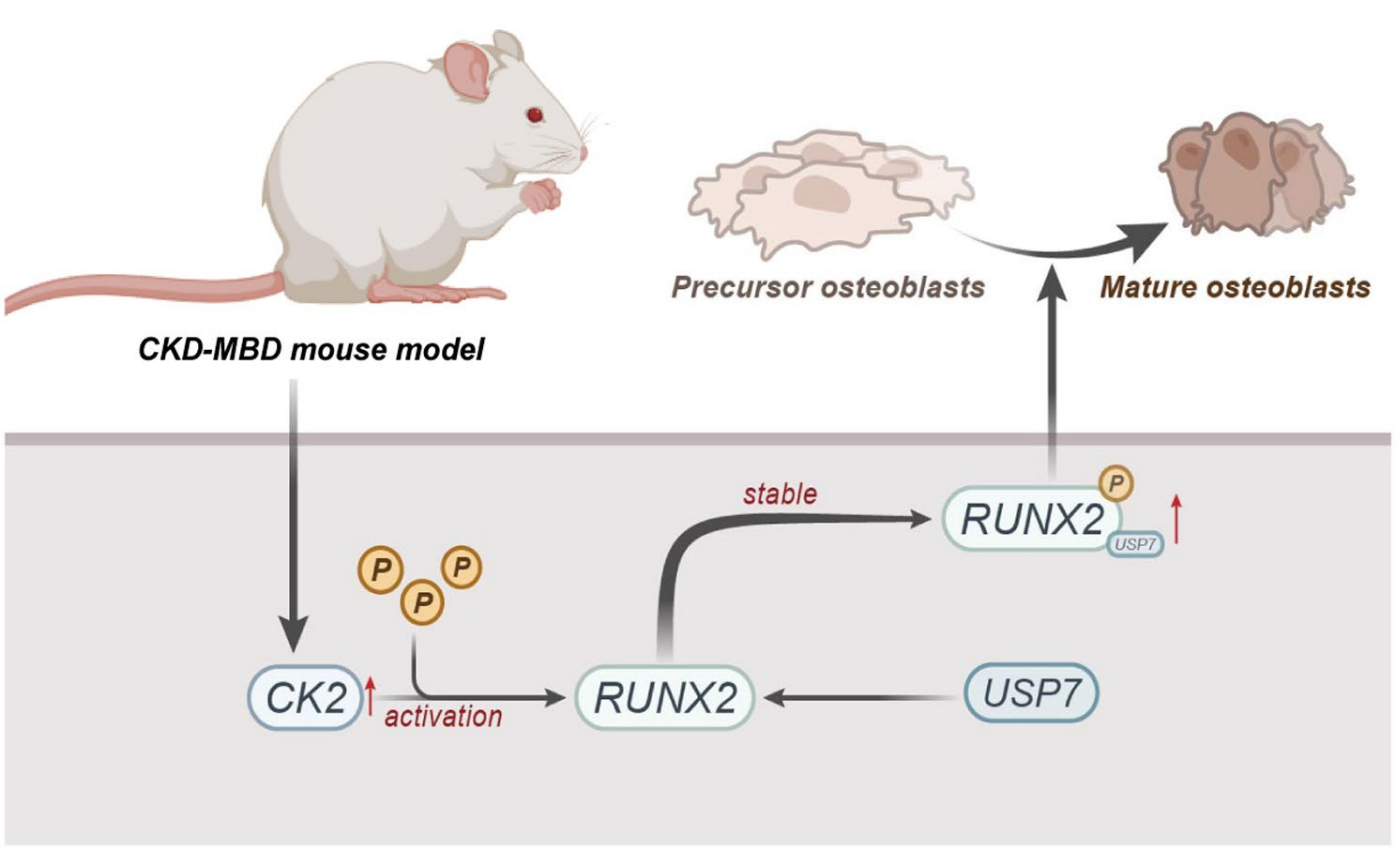
Molecular Mechanism Diagram of CK2 Phosphorylation Modifications on RUNX2 Affecting MBD in CKD Mice

As shown in Fig. 8B, compared to the oe-NC + sh-NC group, the oe-CK2 + sh-NC group mice had significantly increased serum PINP content and decreased CTx expression levels; and compared to the oe-CK2 + sh-NC group, the oe-CK2 + sh-RUNX2 group mice exhibited significantly decreased serum PINP content and increased CTx expression levels (Fig. 7C).

Furthermore, Micro-CT 3D scanning analysis of tibial trabeculae below the proximal growth plate and cortical bones in the midshaft of femurs and tibias revealed that, compared to the oe-NC + sh-NC group, the oe-CK2 + sh-NC group mice displayed higher trabecular bone density and thicker tibial cortical bone; and compared to the oe-CK2 + sh-NC group, the oe-CK2 + sh-RUNX2 group mice showed lower trabecular bone density and thinner tibial cortical bone (Fig. 7D).

The morphometric analysis of pathological tissue showed that compared to the oe-NC + sh-NC group, the mice in the oe-CK2 + sh-NC group exhibited a significant increase in BV/TV, Tb. N, Tb. Th, BV/TV, Ct. Ar, and Ct. Th, along with a decrease in Tb. Sp. In contrast, when compared to the oe-CK2 + sh-NC group, the mice in the oe-CK2 + sh-RUNX2 group displayed a significant reduction in BV/TV, Tb. N, Tb. Th, BV/TV, Ct. Ar, and Ct. Th, while exhibiting an increase in Tb. Sp (Fig. 7E).

The results of MASSON staining revealed that compared to the oe-NC + sh-NC group, the cortical bone tissue volume proportion in mice from the oe-CK2 + sh-NC group significantly increased. Furthermore, compared to the oe-CK2 + sh-NC group, the mice in the oe-CK2 + sh-RUNX2 group exhibited a notable decrease in the cortical bone tissue volume proportion (Fig. 7F-G). Additionally, we found that compared to the sham + ocNC + shNC group, the sham + oeCK2 + shNC group led to an increase in PINP, a decrease in CTx, higher trabecular bone density, thicker tibial cortical bone, and improvements in various histomorphometric parameters (Fig. 7B-G).

These findings indicate that CK2, by regulating the expression of RUNX2, decreases the bone turnover rate in CDK-MBD mice, enhances bone density and strength, and ameliorates Mineral and Bone Disorder in CKD-MBD mice.

## Discussion

CKD induces complex interplays among phosphorus, calcium, and PTH, potentially leading to complications, including fractures, vascular calcifications, and cardiovascular diseases (Bañuelos-Chávez et al. 2017). Common complications in CKD include secondary hyperparathyroidism, characterized by high-turnover renal osteodystrophy, with increased bone turnover leading to bone fragility and an increased risk of fractures, involving disturbances in calcium, phosphorus, PTH, vitamin D, and FGF23 levels (Brandenburg and Ketteler, 2022). This is consistent with the elevated PTH, ALP, and FGF23 levels observed in our CKD-MBD model serum. Elevated PTH and ALP drive abnormal bone remodeling. The increase in PTH and ALP leads to increased osteoclast activity (Oc.N/BS) and bone resorption parameters (E.Pm/B.Pm) (Xie et al. 2023). Despite increased bone turnover, impaired mineralization results in a decrease in bone formation rate (BFR/Ob), which is consistent with the changes in BFR/Ob observed in our CKD-MBD model. CKD is closely associated with bone health, yet the mechanisms underlying skeletal metabolism impairment in CKD remain to be further elucidated (Sugatani 2018). Here, we investigate the role of CK2 in CKD-MBD and elucidate a potential mechanistic basis by which CK2 alleviates CKD-MBD through interactions with RUNX2 and USP7.

Comparison with other bone metabolism disorders: CKD-MBD has unique features compared to other bone metabolism disorders. CKD-related high-turnover bone disease is characterized by increased bone turnover, whereas osteoporosis may involve changes in bone turnover, but not necessarily high turnover. Compared to low-turnover bone disease, CKD-related high-turnover bone disease involves increased bone formation and resorption, whereas low-turnover bone disease shows reduced bone formation and resorption. These features indicate that CKD-related high-turnover bone disease differs from other bone diseases in its pathophysiology, clinical manifestations, and treatment strategies, necessitating targeted management and therapy.

We observed a downregulation of RUNX2 expression in CKD mice. We found that RUNX2 expression was downregulated in CKD mice. The RUNX2 gene has two major transcripts: Runx2-I and Runx2-II (Xiao et al. 2004). Existing studies suggest that both Runx2-I and Runx2-II play important roles in osteogenesis. Runx2-I acts primarily early in osteoblast differentiation (Okura et al. 2014), whereas Runx2-II is mainly expressed in mineralizing bone lining cells and plays a crucial role in bone formation and mineralization (Xiao et al. 2005). Our study examined the expression of both RUNX2 transcripts, showing that RUNX2-II is the primary transcript affecting MBD under CKD conditions. Additionally, silencing of RUNX2 reduced BMD and bone strength in CKD-MBD mice while inhibiting the proliferation and differentiation of osteoblasts. RUNX2 is a key regulatory factor in osteogenesis and may impact vascular calcification in CKD patients (Mizobuchi et al. 2009). Differential expression of RUNX2 was observed in CKD-MBD mice (Liu et al. 2021). Early in CKD, silencing of RUNX2 through ActRIIA leads to MBD components such as osteoporosis and cardiovascular diseases (Williams et al. 2018). Increasing the transcriptional activity of RUNX2 in osteoblast precursor cells through Lmx1b deletion enhances osteoblast differentiation and induction of bone formation (Kim et al. 2022a, b). Reduced mRNA and protein expression of RUNX2 were found in patients with adolescent idiopathic scoliosis (AIS) and decreased BMD (Wang et al. 2014). These findings support our discovery of the role of RUNX2 in alleviating CKD-MBD.

Furthermore, we discovered that USP7 stabilizes RUNX2 expression in CKD-MBD through deubiquitination. As a deubiquitinating enzyme, USP7 eliminates ubiquitin, protecting substrate proteins from degradation (Wang et al. 2019). Studies suggest that another USP member, USP34, stabilizes RUNX2 expression, thereby inducing osteoblast differentiation and bone formation (Guo et al. 2018). In our study, we demonstrate through in vitro deubiquitination assays and ELISA that USP7 stabilizes RUNX2 expression in CKD-MBD through deubiquitination. USP7 is essential for human adipose-derived stem cell osteogenic differentiation and may hold promise in bone tissue engineering (Tang et al. 2017). Moreover, treatment with Eucommia ulmoides leaf extract increased the expression of USP7 and RUNX2, enhancing osteoblast differentiation and mineralization (Guan et al. 2021). Additionally, decreased expression of USP7 was found in tissues of osteoporotic mice, while overexpression of USP7 increased BMD and promoted osteoblast differentiation (Lu et al. 2021). These studies underscore the potential therapeutic role of USP7 in bone-related diseases yet fail to explore the specific role of USP7 in the context of bone diseases induced by CKD.

Our current study demonstrated that CK2 phosphorylates and activates RUNX2, recruiting USP7 to stabilize the expression of RUNX2 in CKD-MBD. Notably, the interaction between CK2 and RUNX2/USP7 has been previously implied. The release of CK2 from different sites of BMPRIa can promote the activation of the BMP signaling pathway (Akkiraju et al. 2016), which is essential for inducing the osteogenic cell phenotype dependent on RUNX2 (Phimphilai et al. 2006); moreover, activated RUNX2 transcription can regulate a BMP member to accelerate osteoblast maturation (Chiou et al. 2011). CK2 can bring functional activation of USP7 in chronic lymphocytic leukemia (Carrà et al. 2017). Previous studies have confirmed that CK2 phosphorylates RUNX2 to recruit USP7, stabilizing RUNX2 expression and inducing bone formation (Kim et al. 2020). Our research also confirms the regulatory role of the CK2/USP7/RUNX2 axis in bone metabolism, but further reveals its regulatory role in CKD-induced MBD. We further elucidate that CK2 can phosphorylate RUNX2, activate it, and recruit USP7 to mediate deubiquitination, stabilizing RUNX2 expression and improving osteogenesis and bone metabolism in the CKD-MBD model. Moreover, our bioinformatics analysis confirmed the critical genetic roles of RUNX2 and USP7 in CKD-MBD. Increasing evidence indicates that CK2 has regulatory functions in bone diseases. Interestingly, CK2 activation induced by AsA stimulates osteoblast differentiation by increasing ALP activity and decreasing Ikaros activity (Son et al. 2007). Increasing evidence suggests that CK2 plays a regulatory role in bone diseases. Intriguingly, AsA-induced CK2 activation stimulates osteoblast differentiation by increasing ALP activity and reducing Ikaros activity (Son et al. 2007). The lack of regulation of CK2 subunit (Csnk2b) in bone cells may lead to decreased bone mass (Kim et al. 2022a, b). Previous predictions through mathematical models suggest that CK2.3 enhances the mineralization of osteoporotic bones (Lisberg et al. 2017). Studies have also indicated that stimulation of CK2.3 increases bone sialoprotein and ALP expression in osteoblasts (Weidner et al. 2019). RUNX2 and CK2 pathways play important roles in CKD-MBD, and their dysregulation exacerbates bone metabolism abnormalities in CKD. RUNX2 is a critical transcription factor essential for osteoblast differentiation, and it regulates osteoblast differentiation via BMP4 signaling. RUNX2 downregulation impairs osteoblast differentiation (Liu et al. 2022; Gargalionis et al. 2024). CK2 stabilizes RUNX2 by phosphorylating it and recruiting USP7. Disruption of CK2 signaling affects RUNX2 stability and activity, weakening osteogenesis. Our study reveals that CK2 promotes the proliferation and differentiation of osteoblast precursor cells, achieved through interactions with RUNX2 and USP7.

In conclusion, this study demonstrates that CK2, by phosphorylating and activating RUNX2, recruiting USP7 to stabilize RUNX2 expression, promotes the differentiation of osteoblast precursor cells into mature osteoblasts, thereby alleviating MBD in CKD mice (Fig. 8). This finding may provide a new direction for understanding the molecular mechanisms of CKD-MBD. These findings could serve as a reference for future research on developing novel therapeutic strategies for CKD-MBD, aiming to improve the quality of life of CKD patients and reduce morbidity and mortality. However, it is important to note that the CKD-MBD animal model used in this study may have limitations in representing the complex human disease state, warranting further research to validate the translational relevance of these findings.

## Electronic supplementary material

Below is the link to the electronic supplementary material.

Supplementary Material 1: Fig. S1: Validation of Different Silencing Sequences Efficiency. Note: (A) RT-qPCR and Western Blot validation of the efficiency of three silencing sequences for RUNX2; (B) RT-qPCR and Western Blot validation of the efficiency of three silencing sequences for USP7; (C) RT-qPCR and Western Blot validation of the efficiency of three silencing sequences for CK2; * indicates significance compared to the sh-NC group at *P* < 0.05; cellular experiments were repeated three times

Supplementary Material 2: Figure S2. Bioinformatics analysis to identify key genes involved in CKDNote: (A) Clustering dendrogram of 18 samples; (B) The scale-free index (left) and the average connectivity (right) for various soft threshold powers, with the red line indicating the correlation coefficient; (C) Clustering dendrogram of co-expressed genes, where each leaf represents a distinct gene module; (D) Heatmap showing the correlation between modules and traits in the control and CKD groups, with each cell containing the corresponding correlation and P-value; (E) Venn diagram of the intersection of MEred module characteristic genes and DEGs.

Supplementary Material 3: Figure S3. Effects of Silenced or Overexpressed RUNX2 on Proliferation and Differentiation of Osteogenic Precursor Cells. Note: (A) Detection of relative expression levels of RUNX2 in various cell groups by RT-qPCR; (B) CCK-8 assay for assessing cell viability in different groups; (C) Detection of relative expression levels of ALP, Collagen-1, and Osteocalcin in various cell groups by RT-qPCR; * indicates statistical significance between two groups, P < 0.05; all cell experiments were repeated three times.

Supplementary Material 4

## Data Availability

The datasets generated and analyzed during the current study are available from the corresponding author on reasonable request.

## Abbreviations

CKD: Chronic Kidney Disease
MBD: Mineral Bone Disorder
RUNX2: Runt-Related Transcription Factor 2
USP7: Ubiquitin-Specific Protease 7
CK2: Casein Kinase 2
PTH: Parathyroid Hormone
FGF23: Fibroblast Growth Factor 23
ALP: Alkaline Phosphatase
BMD: Bone Mineral Density
BMP: Bone Morphogenetic Protein
E.Pm/B.Pm: Eroded Perimeter per Bone Perimeter
Oc.N/BS: Osteoclast Number per Bone Surface
BFR/Ob: Bone Formation Rate per Osteoblast Surface
BV/TV: Bone Volume Fraction
Tb. N: Trabecular Number; Tb
Th: Trabecular Thickness
Tb. Sp: Trabecular Separation
Ct. Ar: Cortical Area
Ct. Th: Cortical Thickness
RT-qPCR: Reverse Transcription Quantitative Polymerase Chain Reaction
ELISA: Enzyme-Linked Immunosorbent Assay
