# Supplementary figures and images for: Targeting casein kinase 2 and ubiquitin-specific protease 7 to modulate RUNX2-mediated osteogenesis in chronic kidney disease
Source: Mol Med. 2025 May 30;31:214. doi: 10.1186/s10020-025-01222-5 (PMC12125883; doi:10.1186/s10020-025-01222-5)

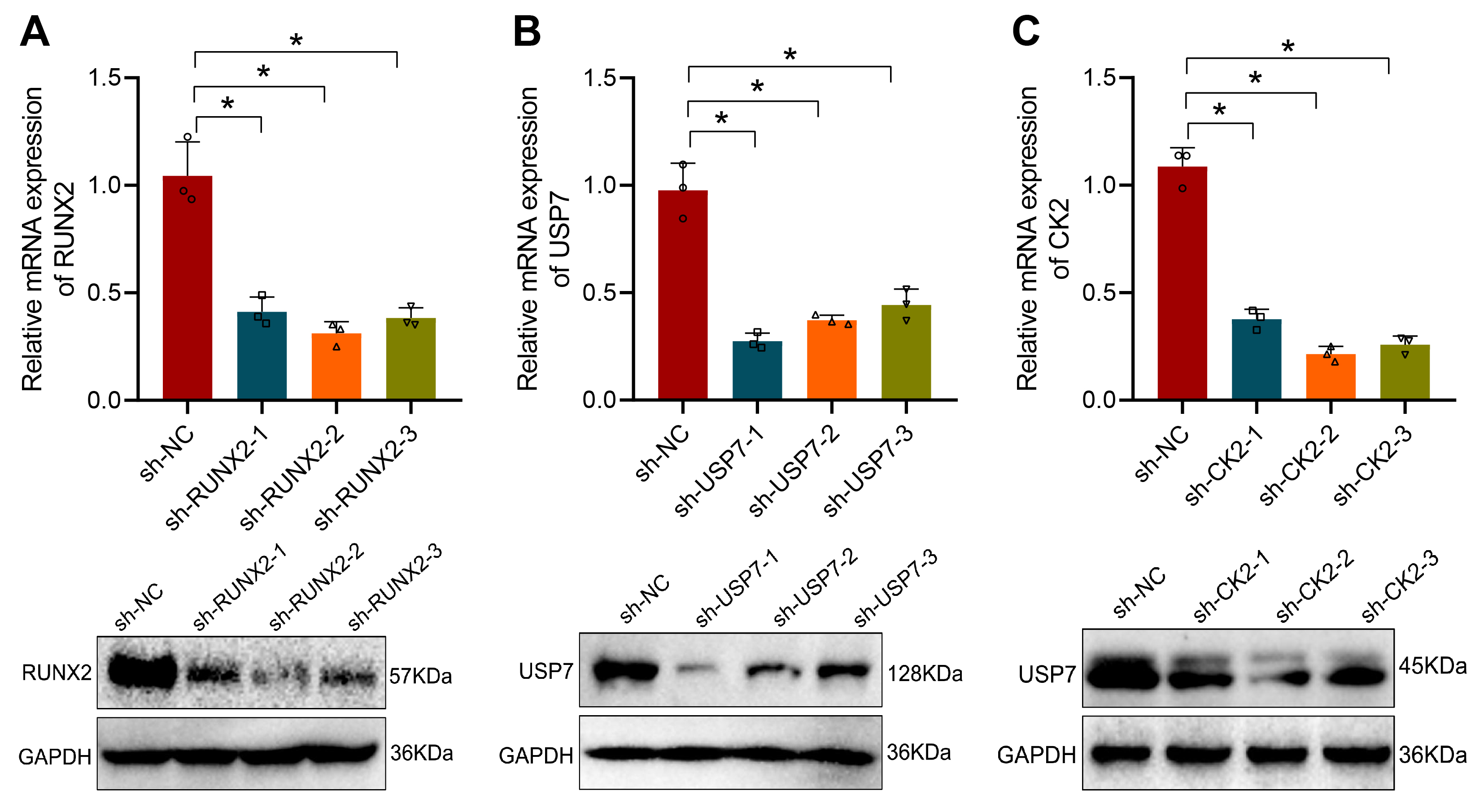

Supplement: Supplementary file 1 — Supplementary Material 1: Fig. S1: Validation of Different Silencing Sequences Efficiency. Note: (A) RT-qPCR and Western Blot validation of the efficiency of three silencing sequences for RUNX2; (B) RT-qPCR and Western Blot validation of the efficiency of three silencing sequences for USP7; (C) RT-qPCR and Western Blot validation of the efficiency of three silencing sequences for CK2; * indicates significance compared to the sh-NC group at P < 0.05; cellular experiments were repeated three times [file 10020_2025_1222_MOESM1_ESM.jpg]

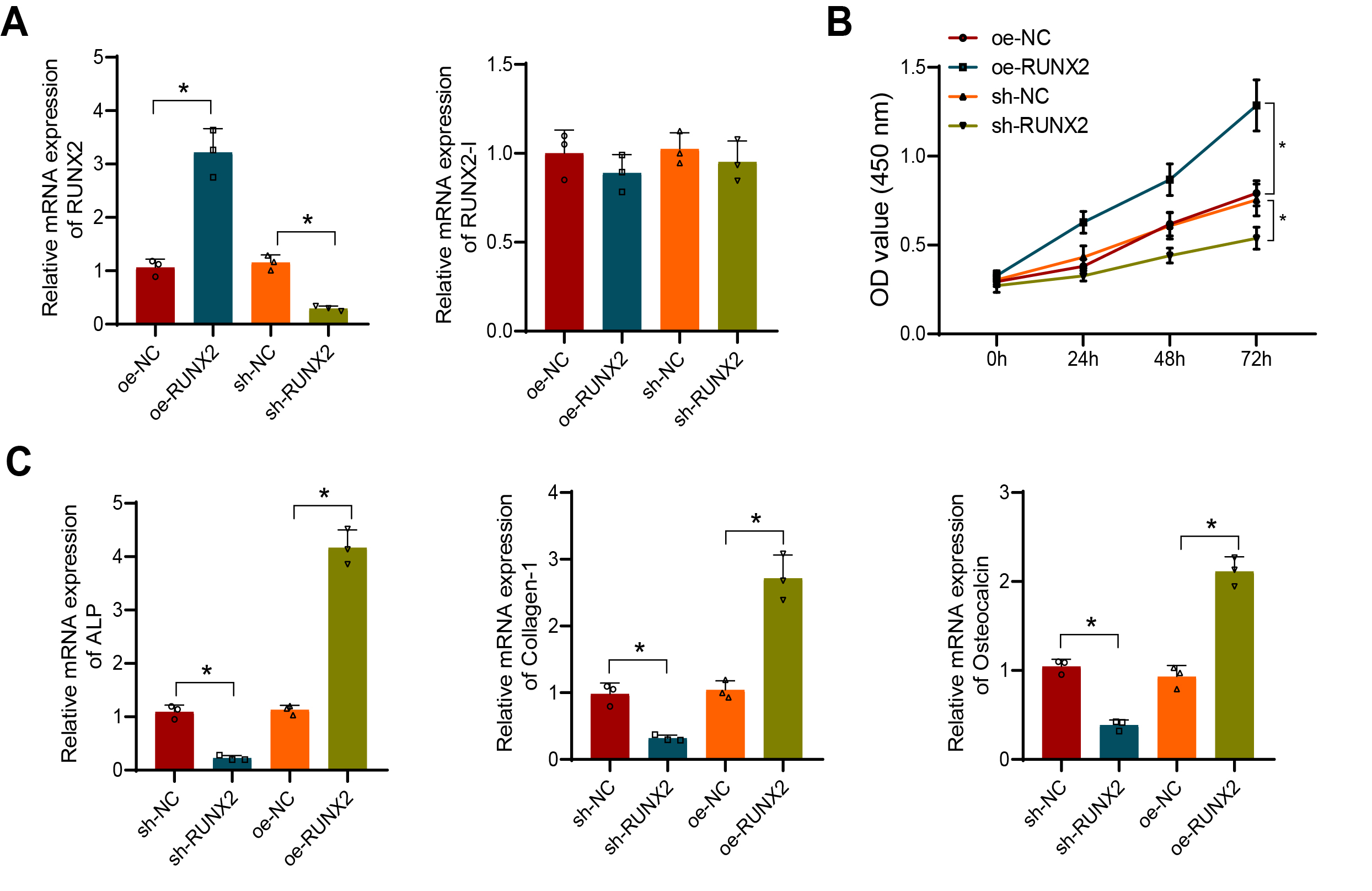

Supplement: Supplementary file 2 — Supplementary Material 2: Figure S2. Bioinformatics analysis to identify key genes involved in CKDNote: (A) Clustering dendrogram of 18 samples; (B) The scale-free index (left) and the average connectivity (right) for various soft threshold powers, with the red line indicating the correlation coefficient; (C) Clustering dendrogram of co-expressed genes, where each leaf represents a distinct gene module; (D) Heatmap showing the correlation between modules and traits in the control and CKD groups, with each cell containing the corresponding correlation and P-value; (E) Venn diagram of the intersection of MEred module characteristic genes and DEGs. [file 10020_2025_1222_MOESM2_ESM.jpg]

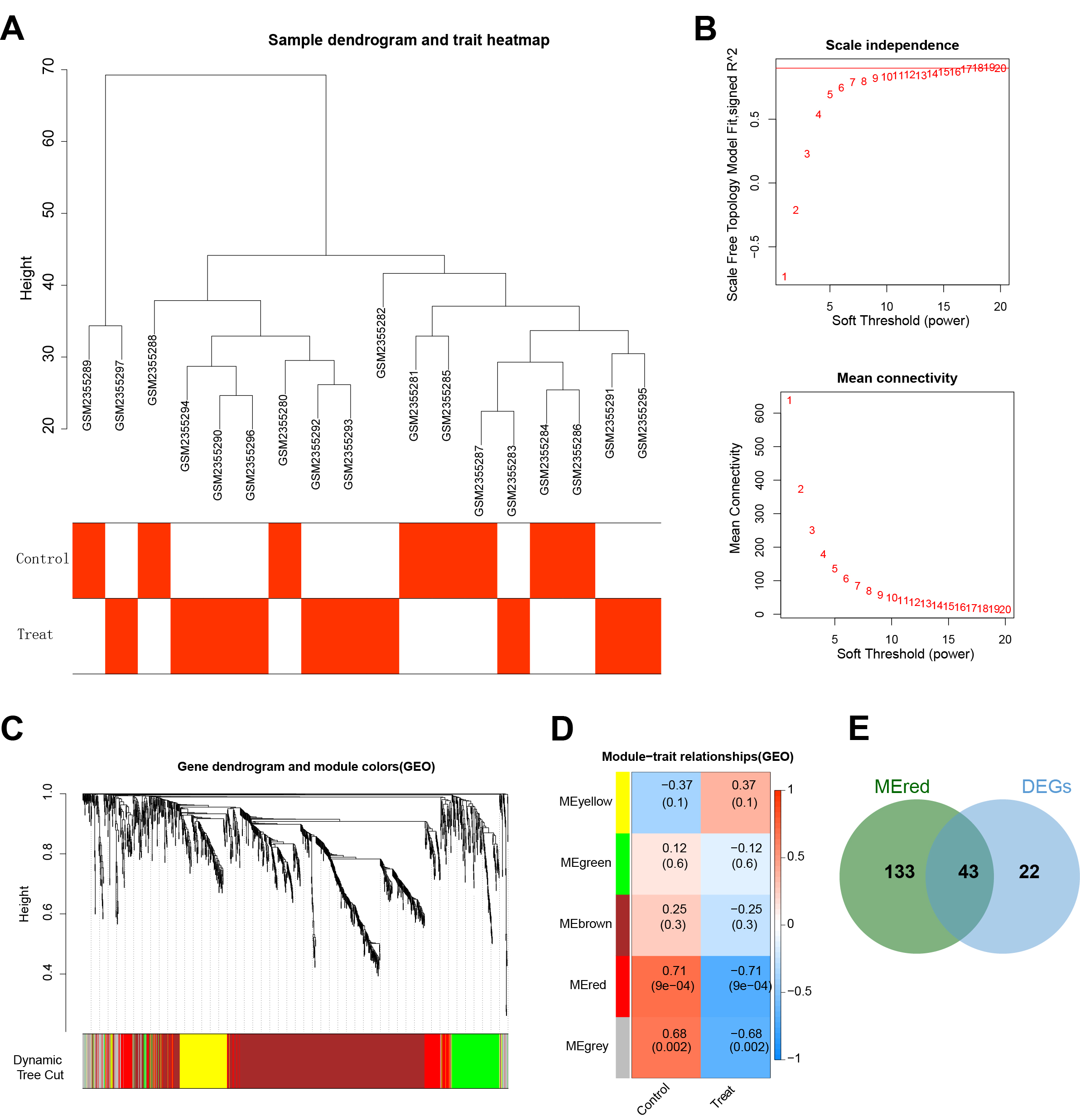

Supplement: Supplementary file 3 — Supplementary Material 3: Figure S3. Effects of Silenced or Overexpressed RUNX2 on Proliferation and Differentiation of Osteogenic Precursor Cells. Note: (A) Detection of relative expression levels of RUNX2 in various cell groups by RT-qPCR; (B) CCK-8 assay for assessing cell viability in different groups; (C) Detection of relative expression levels of ALP, Collagen-1, and Osteocalcin in various cell groups by RT-qPCR; * indicates statistical significance between two groups, P < 0.05; all cell experiments were repeated three times. [file 10020_2025_1222_MOESM3_ESM.jpg]
