# Supplementary material for: Targeting casein kinase 2 and ubiquitin-specific protease 7 to modulate RUNX2-mediated osteogenesis in chronic kidney disease
Source: Mol Med. 2025 May 30;31:214. doi: 10.1186/s10020-025-01222-5 (PMC12125883; doi:10.1186/s10020-025-01222-5)
Supplement: Supplementary file 4 — Supplementary Material 4 [file 10020_2025_1222_MOESM4_ESM.docx]

**Table S1. Lentiviral vectors expressing shRNA sequences.**

| **shRNA** | **Sequence (5'-3')** |
| --- | --- |
| sh-NC | 5'-CCTAAGGTTAAGTCGCCCTCG-3' |
| sh-RUNX2-1 | 5'-GCACGCTATTAAATCCAAATT-3' |
| sh-RUNX2-2 | 5'-GCAGAATGGATGAGTCTGTTT-3' |
| sh-RUNX2-3 | 5'-GAGTTTCACCTTGACCATAAC-3' |
| sh-USP7-1 | 5'-TGCGAAATCTGCCATGGAA-3' |
| sh-USP7-2 | 5'-CTCAGAACCCTGTGATCAA-3' |
| sh-CK2-1 | 5'- GCTGCATTTAGGTGGAGACTT -3' |
| sh-CK2-2 | 5'- CGTAAACAACACAGACTTCAA -3' |
| sh-CK2-3 | 5'- CAAGAATATAATGTCCGAGTT -3' |

Note: sh-, short hairpin RNA-; NC, negative control.

**Table S2. Primer sequences for RT-qPCR (mouse).**

| **Gene** | **Sequence** |  |
| --- | --- | --- |
| ALP | Forward: 5′- ACCACCACGAGAGTGAACCA -3′ |  |
|  | Reverse: 5′- CGTTGTCTGAGTACCAGTCCC -3′ |  |
| Collagen-1 | Forward: 5′-TTCTCCTGGCAAAGACGGAC-3′ |  |
|  | Reverse: 5′-CCATCGGTCATGCTCTCTCC-3′ |  |
| Osteocalcin | Forward: 5ʹ- CGCTACCTGTATCAATGGCTGG -3' |  |
|  | Reverse: 5ʹ- CTCCTGAAAGCCGATGTGGTCA -3' |  |
| RUNX2-II | Forward: 5′- TGGTTACTGTCATGGCGGGTA -3′ |  |
|  | Reverse: 5′- TCTCAGATCGTTGAACCTTGCTA -3′ |  |
| RUNX2-I | Forward: 5′- CGACAGTCCCAACTTCCTGT-3′ | |
|  | Reverse: 5′- CGGTAACCACAGTCCCATC-3′ | |
| USP7 | Forward: 5′-GTCACGATGACGACCTGTCTGT-3′ |  |
|  | Reverse: 5′-GTAATCGCTCCACCAACTGCTG-3′ |  |
| GAPDH | Forward: 5′-GTCTCCTCTGACTTCAACAGCG-3′ |  |
|  | Reverse: 5′-ACCACCCTGTTGCTGTAGCCAA-3′ |  |

Note: RT-qPCR, reverse transcription-quantitative polymerase chain reaction.
